# Influence of Polyunsaturated Fatty Acid Intake on Kidney Functions of Rats with Chronic Renal Failure

**DOI:** 10.3390/md19120692

**Published:** 2021-12-03

**Authors:** Hiroki Muramatsu, Naoe Akimoto, Michio Hashimoto, Kenji Sugibayashi, Masanori Katakura

**Affiliations:** 1 Laboratory of Nutritional Physiology, Department of Pharmaceutical Sciences, Faculty of Pharmacy and Pharmaceutical Sciences, Josai University, Sakado 350-0295, Saitama, Japan; gkd2001@josai.ac.jp (H.M.); akimoe@josai.ac.jp (N.A.); sugib@josai.ac.jp (K.S.); 2 Faculty of Medicine, Shimane University, Izumo 693-8501, Shimane, Japan; michio1@med.shimane-u.ac.jp

**Keywords:** chronic kidney disease, ARA, DHA, urinary albumin, oxidative stress

## Abstract

Arachidonic acid (ARA), an omega-6 (ω-6) polyunsaturated fatty acid (PUFA), is involved in the development and maintenance of renal functions, whereas docosahexaenoic acid (DHA) is an omega-3 (ω-3) PUFA that has anti-inflammatory effects and attenuates nephropathy. However, their effects on the progression of chronic kidney disease (CKD) remain unknown. The aim of this study was to assess the effects of feeding ARA, DHA, and ARA and DHA-containing diets on rats with 5/6 nephrectomized kidneys. Urine and feces were collected every 4 weeks, and the kidneys were collected at 16 weeks after surgery. Urinary albumin (U-ALB) excretion increased gradually with nephrectomy, but the U-ALB excretion was attenuated by feeding the rats with an ARA + DHA-containing diet. Reactive oxygen species (ROS) levels in the kidneys were lower in the ARA + DHA group than in the other groups. At 4 weeks after surgery, the lipid peroxide (LPO) levels in the plasma of the ARA + DHA groups decreased significantly after surgery compared to the control CKD group, but this did not happen at 16 weeks post-surgery. There was a significant negative correlation between LPO levels in the plasma at 4 weeks and creatinine clearance, and a positive correlation with urinary albumin levels. These results suggest that the combination of ARA and DHA inhibit the progress of early stage CKD.

## 1. Introduction

The kidneys play an important role in the homeostasis of body fluids (the balance of water, salts, and minerals such as sodium, calcium, phosphorus, and potassium in the blood), regulation of blood pressure, production of red blood cells, and in maintaining bones strength.

Blood pressure increases with age. Water, salt, and sodium concentration in the body increases due to decreased kidney functions, resulting in increased blood pressure. Further, the blood volume increases and the blood vessels become hard or clogged, making them more susceptible to arteriosclerosis [1]. Moreover, the number of glomeruli decreases with age, resulting in a decreased glomerular filtration rate (GFR). Chronic kidney disease (CKD) occurs when albuminuria and a GFR of <60 mL/min/1.73 m^2^ persists for 3 months or more. Kidney disease progression is assessed by changes in the GFR stage. The cause of CKD is complications of lifestyle-related diseases such as diabetes, high blood pressure, and obesity. The longer a person suffers from these diseases, the more likely they are to develop CKD. Diabetic nephropathy, chronic glomerulonephritis, and nephrosclerosis are the most common causes of kidney failure in Japan. Kidneys cannot be regenerated using the current medical technology. Therefore, loss of kidney function cannot be reversed and patients require kidney transplantation or dialysis as a treatment. Patients on dialysis must follow a strict diet and face daily restrictions because they need to visit the hospital several times a week, which leads to a decrease in the activities of daily living (ADL). The number of patients undergoing dialysis in Japan has increased compared to those in Western countries. Patients being treated with dialysis have increased by 1.9% since 2016; thus, currently there are over 300 thousand dialysis patients, and the health expenditures have increased by over 1 trillion yen. Consequently, this increase has become a social problem. Therefore, it is important to establish a method to delay the progression of chronic renal failure [2].

Polyunsaturated fatty acids (PUFAs) are important for the development and maintenance of normal renal functions. Fatty acids are nutrients that are consumed daily, which act not only as an energy source but also help to carry out various biological reactions through receptors and other binding proteins. The intake of PUFAs is necessary because they are difficult to biosynthesize in sufficient amounts in vivo. It has become clear that there are abnormalities in their biological function and due to insufficient intake of PUFAs. Arachidonic acids (ARA), omega-6 (ω-6) PUFAs, are involved in the development and maintenance of normal renal functions. The active molecule prostaglandin (PG) is being produced from ARA by enzymes such as cyclooxygenase (COX), a rate-limiting enzyme, in the pathway of synthesizing PG from ARA, and lipoxygenase (LOX). PGs adjust the blood flow to the kidneys, the amount of body water, and the balance of sodium concentration [3]. Docosahexaenoic acid (DHA) and/or eicosapentaenoic acid (EPA) are omega-3 (ω-3) PUFAs that protect against ischemic acute renal failure [4], IgA or cyclosporine A-induced nephrotoxicity [5,6,7,8,9], streptozocin-induced type 1 diabetes [10], and type 2 diabetic nephropathy in rodents [11,12,13,14]. These types of renal failures are closely related to inflammation, which promotes the loss of renal function [3]. DHA and EPA are oxidized by COXs, LOXs, or cytochrome P450 monooxygenases to produce DHA-derived docosanoids and EPA-derived eicosanoids, which have anti-inflammatory effects [15]. Dietary ω-3 fatty acids are considered to prevent inflammation through a variety of activities linked to the inhibition of ARA-derived eicosanoid-mediated effects, anti-inflammatory properties, and competitive inhibition of cytokines and ARA-derived eicosanoid synthetic enzymes [3]. However, there are no available studies on the combination of ARA and DHA or the direct comparison between them.

Chronic inflammation and oxidative stress lead to the progression of chronic renal failure. Under normal physiological conditions, the oxygen free radicals and the antioxidant defense mechanisms are balanced. Disturbance in the balance between the pro-oxidants and antioxidants results in oxidative stress in pathological conditions, leading to cellular damage. Oxidative stress is known to increase cytokine production [16].

The aim of this study was to explore the effects of different kinds of dietary ω-3 and ω-6 PUFAs on the progression of CKD using nephrectomized rats that had lost five-sixths of their kidneys, and the effect of PUFA intake on oxidative stress and inflammation in the kidney. In this study, we assessed renal functions, such as creatinine clearance and urinary albumin. Kidney weight, histological evaluation, and protein and triglyceride levels were used to assess the renal status. Oxidative stress parameters, such as reactive oxygen species (ROS) and peroxynitrate (ONOO^−^) in the kidney, and lipid peroxide (LPO) in the blood and kidney were also assessed. Cytokines such as interleukin (IL)-6, tumor necrosis factor (TNF)-α, and transforming growth factor (TGF)-β_1_ in the kidney were assessed as a measures of kidney inflammation.

## 2. Results

### 2.1. Body and Kidney Weight

**Figure 1:**
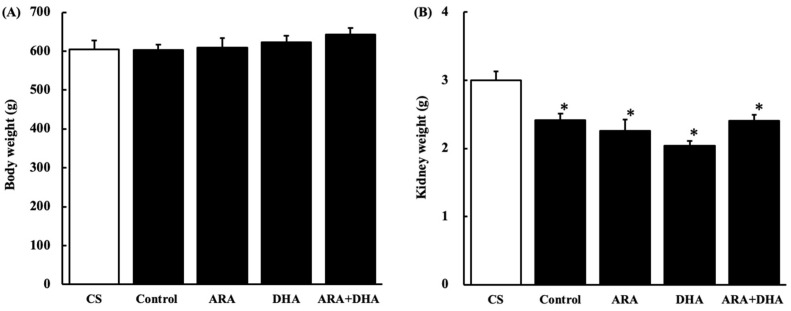
Body weight and kidney weight at 16 weeks after nephrectomy. (**A**) body weight; (**B**) kidney weight. The value in CS group represents two kidneys; the value in all other groups is the remaining kidney after nephrectomy of the five sixths of the kidneys. Values are presented as the mean ± SEM (*n* = 6–9). * *p* < 0.05, by ANOVA and Tukey HSD (versus CS group).

Figure 1 shows the body and kidney weight at 16 weeks after nephrectomy (Figure 1A,B). Nephrectomy did not affect the body weight of rats, as there were no significant differences among the five groups. However, kidney weight was significantly decreased by nephrectomy.

### 2.2. Renal Parameter

Blood and urine were collected at 0, 4, 8, and 12 weeks after nephrectomy to assess blood and urinary glucoses and renal function. Table 1 shows that changes of blood glucose, urinary glucose, creatinine clearance, and urinary albumin. The levels of blood and urinary glucose were not significantly different among the five groups. Creatinine clearance in the control sham (CS) group gradually decreased and significantly decreased by nephrectomy. Urinary albumin increased significantly but it was attenuated in the ARA + DHA group.

### 2.3. Fatty Acid Composition of Total Lipids in the Plasma and Kidneys

Table 2 shows fatty acid composition of total lipid in plasma at 16 weeks after nephrectomy. The main fatty acids in the plasma are C16:0, C16:1, C18:0, C18:1, C18:2ω-6, C20:0, C20:4ω-6, C20:5ω-3, C22:0, C22:6ω-3. The increased level of ARA or DHA in kidney was reflective of the specific food diet. DHA levels in the ARA group significantly decreased compared with the control group. The linoleic acid level was significantly decreased in the ARA or ARA + DHA groups compared with that in the control group.

Table 3 shows the fatty acid composition of total lipid in kidney at 16 weeks after nephrectomy. Main fatty acids in the kidney were C8:0, C10:0, C12:0, C14:0, C16:0, C16:1, C18:0, C18:1, C18:2ω-6, C18:3ω-3, C20:4ω-6, C22:0, C24:0, C22:6ω-3. The increased level of ARA or DHA in the kidney was reflective of the specific food diet. Linoleic acid level was significantly decreased in the ARA or ARA + DHA groups compared with that in the control group.

### 2.4. Histological Evaluation

#### 2.4.1. Hematoxylin and Eosin (H&E) Staining

**Figure 2:**
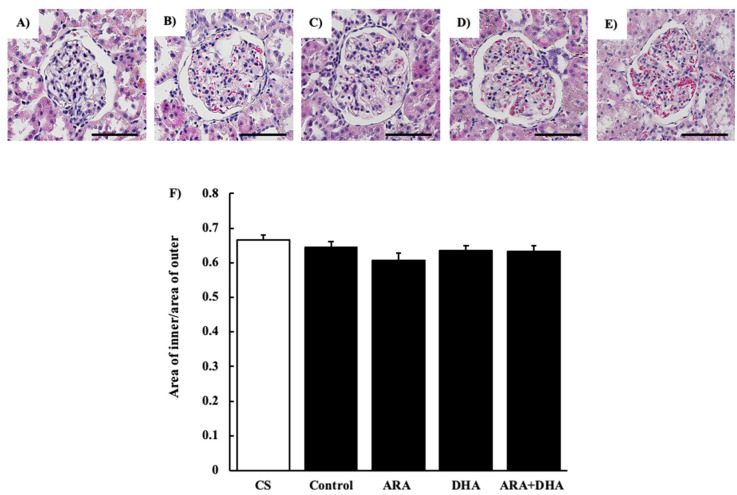
H&E staining of glomeruli at 16 weeks after nephrectomy. Images of coronal sections of the glomeruli from (**A**) CS group; (**B**) control group; (**C**) ARA group; (**D**) DHA group, and (**E**) ARA + DHA group. (**F**) The area of inner/area of outer level was evaluated. Scale bar: 50 µm. Values are presented as the mean ± SEM (*n* = 6–9). There is no significant difference among five groups.

Figure 2 shows the glomerular image (Figure 2A–E) and the area of inner/area of outer level by H&E staining at 16 weeks after nephrectomy (Figure 2F). The ratio of area of inner/area of outer was not significantly different among the five groups.

#### 2.4.2. Periodic Acid Schiff (PAS) Staining

**Figure 3:**
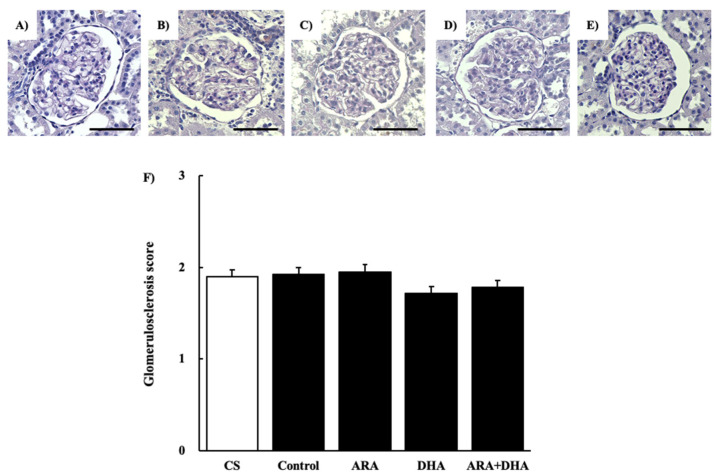
PAS staining of glomeruli at 16 weeks after nephrectomy. Images of coronal sections of the glomeruli from (**A**) CS group; (**B**) control group; (**C**) ARA group; (**D**) DHA group, and (**E**) ARA + DHA group. (**F**) Glomerular sclerosis level was evaluated. Values are presented as the mean ± SEM (*n* = 6–9). Scale bar: 50 µm. There is no significant difference among five groups.

Figure 3 shows the glomerular image (Figure 3A–E) and the glomerular sclerosis level by PAS staining at 16 weeks after nephrectomy (Figure 3F). The glomerular sclerosis level was not significantly different among the five groups.

#### 2.4.3. Periodic Acid Methenamine-Silver (PAM) Staining

**Figure 4:**
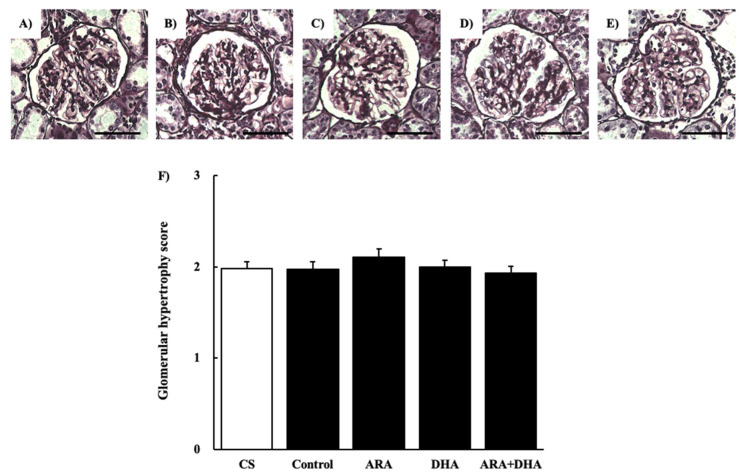
PAM staining of glomeruli at 16 weeks after nephrectomy. Images of coronal sections of the glomeruli from (**A**) CS group; (**B**) control group; (**C**) ARA group; (**D**) DHA group, and (**E**) ARA + DHA group. (**F**) Glomerular hypertrophy level was evaluated. Values are presented as the mean ± SEM (*n* = 6–9) Scale bar: 50 µm. There is no significant difference among five groups.

Figure 4 shows the glomerular images (Figure 4A–E) and the glomerular hypertrophy level by PAM staining of glomeruli at 16 weeks after nephrectomy (Figure 4F). The glomerular hypertrophy level was not significantly different among the five groups.

### 2.5. Protein Levels in Kidney

**Figure 5:**
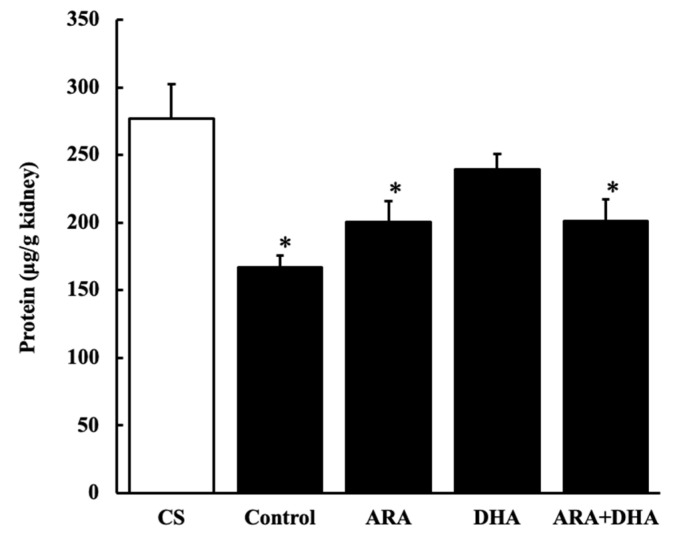
Protein levels in kidney at 16 weeks after nephrectomy. Values are presented as the mean ± SEM (*n* = 6–9). * *p* < 0.05, by ANOVA and Tukey HSD (versus CS group).

Figure 5 shows the protein level in the kidney at 16 weeks after nephrectomy (Figure 5). Protein level in the kidney was significantly decreased after nephrectomy. However, they were not significantly different among the five groups.

### 2.6. Triglycerides Levels in the Kidney 

**Figure 6:**
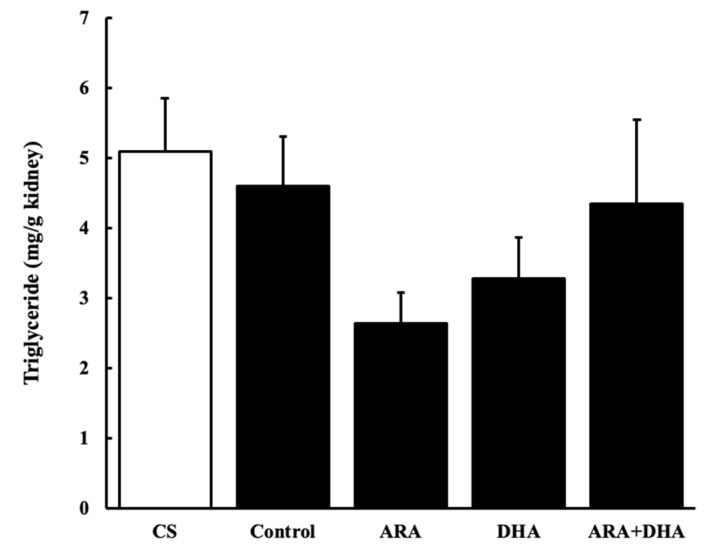
Triglycerides level in kidney at 16 weeks after nephrectomy (Figure 6). Values are presented as the mean ± SEM (*n* = 6–9). There are no significant differences among five groups.

Figure 6 shows triglycerides level in the kidney at 16 weeks after nephrectomy (Figure 6). Triglycerides level in the kidney was not significantly different among the five groups.

### 2.7. Oxidative Stress Status of the Kidney

#### 2.7.1. Reactive Oxygen Species (ROS) Level

**Figure 7:**
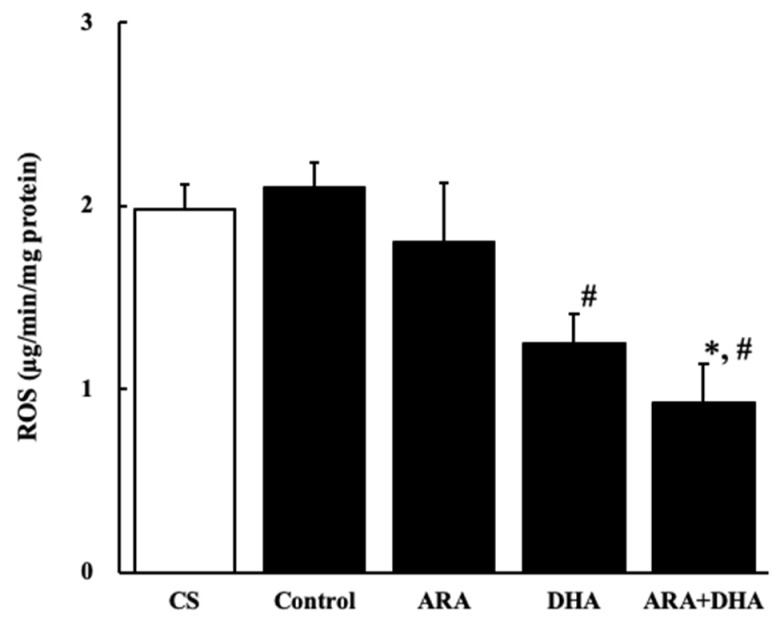
ROS levels in kidney at 16 weeks after nephrectomy. Values are presented as the mean ± SEM (*n* = 6–9). * *p* < 0.01, by ANOVA and Tukey HSD (versus CS and control group). ^#^ *p* < 0.05, by ANOVA and Tukey HSD (versus control group).

Figure 7 shows the ROS level in the kidney at 16 weeks after nephrectomy (Figure 7). ROS level was significantly decreased in the DHA and ARA + DHA groups.

#### 2.7.2. Peroxynitrate (ONOO^−^) Level

**Figure 8:**
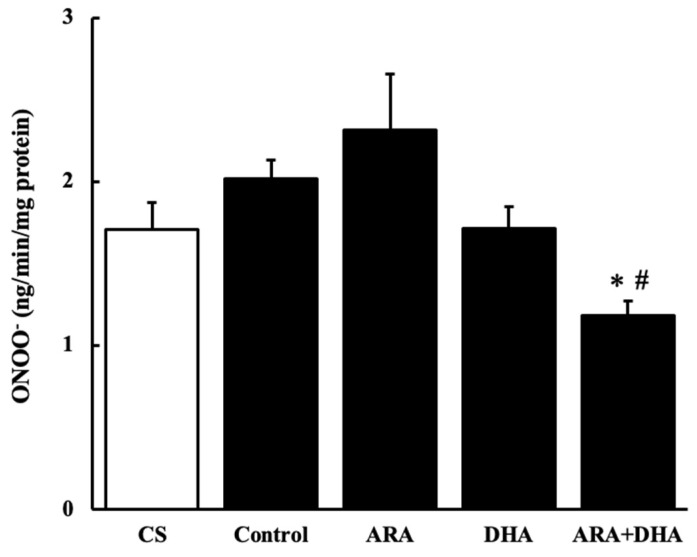
ONOO^−^ levels in kidney at 16 weeks after nephrectomy. Values are presented as the mean ± SEM (*n* = 6–9). * *p* < 0.01, by ANOVA and Tukey HSD (versus ARA group). ^#^ *p* < 0.05, by ANOVA and Tukey HSD (versus control group).

Figure 8 shows the ONOO^−^ level in the kidney at 16 weeks after nephrectomy (Figure 8). The ONOO^−^ level was significantly decreased in the ARA + DHA groups.

#### 2.7.3. Lipid Peroxides (LPO) Level in the Kidney and Plasma

**Figure 9:**
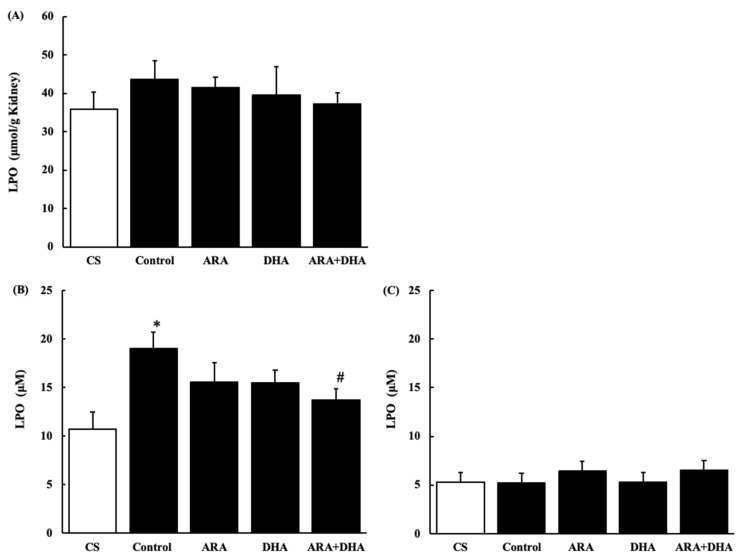
LPO levels in kidney and plasma at 4 and 16 weeks after nephrectomy. (**A**) in the kidney at 16 weeks; (**B**) in plasma at 4 weeks, and (**C**) plasma at 16 weeks. Values are presented as the mean ± SEM (*n* = 6–9). * *p* < 0.05, by ANOVA and Tukey HSD (versus CS group). # *p* < 0.05, by ANOVA and Tukey HSD (versus control group).

Figure 9 shows LPO levels in the kidney and plasma at 4 and 16 weeks after nephrectomy (Figure 9A–C). LPO levels in the kidney showed no significant differences among the five groups. LPO level in plasma was significantly increased after 4 weeks, but it was attenuated in the ARA + DHA group. Interestingly, there were no significant differences among the five groups after 16 weeks of nephrectomy.

### 2.8. Cytokine Levels in the Kidney

**Figure 10:**
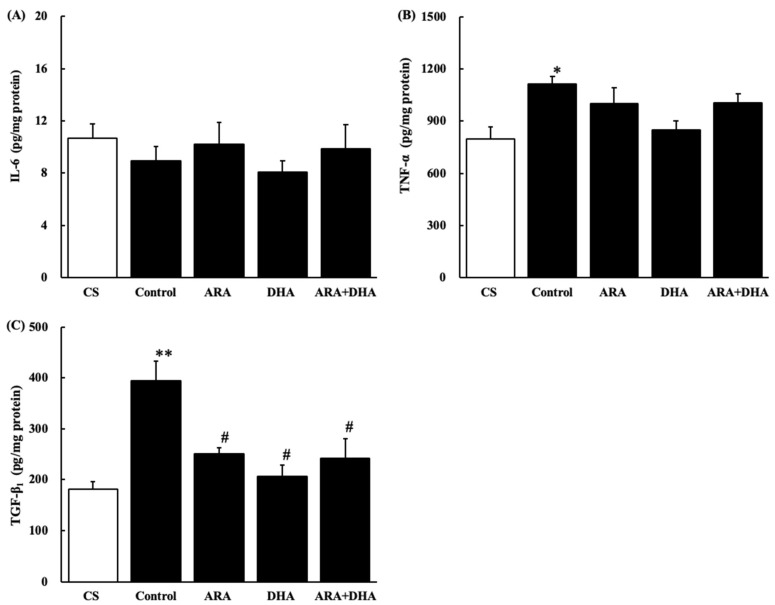
Cytokine levels in the kidney. (**A**) IL-6, (**B**) TNF-α and (**C**) TGF-β_1_. Values are presented as the mean ± SEM (*n* = 6–9). * *p* < 0.05, by ANOVA and Tukey HSD (versus CS group). ** *p* < 0.0001, by ANOVA and Tukey HSD (versus CS group). ^#^ *p* < 0.01, by ANOVA and Tukey HSD (versus control group).

Figure 10 shows cytokine levels in the kidney at 16 weeks after nephrectomy (Figure 10A–C). IL-6 level was not significantly different among the five groups at 16 weeks after nephrectomy. TNF-α and TGF-β_1_ levels were significantly increased after nephrectomy, but TGF-β_1_ levels were significantly decreased in the ARA, DHA, and ARA + DHA groups.

### 2.9. Correlations between Renal Functions and LPO Levels in Plasma

**Figure 11:**
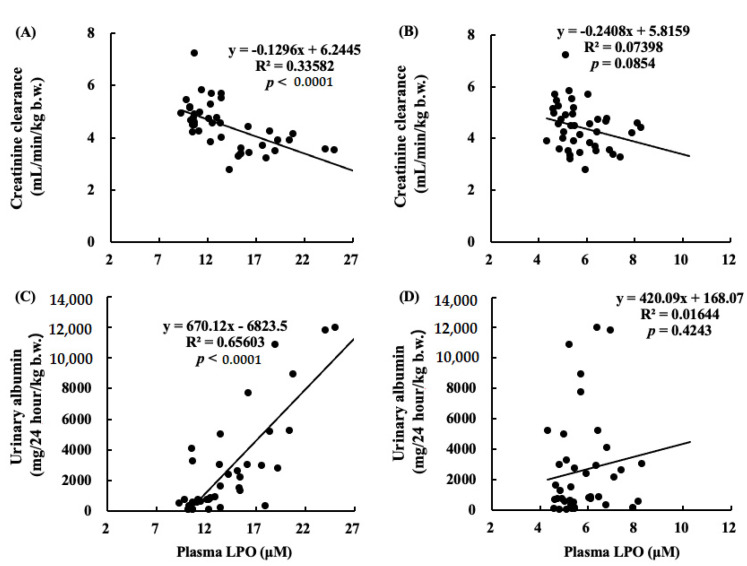
Correlations between C_cr_ and LPO in plasma at (**A**) at 4 weeks or (**B**) at 16 weeks. The negative correlation between C_cr_ and LPO in plasma at 4 weeks (*p* < 0.0001). Correlations between urinary albumin at 12 weeks and LPO in plasma at (**C**) at 4 weeks or (**D**) at 16 weeks. The positive correlation between urinary albumin at 12 weeks and LPO in plasma at 4 weeks (*p* < 0.0001).

Figure 11 shows correlations between C_cr_ and LPO levels in plasma (Figure 11A,B), and urinary albumin and LPO levels in plasma (Figure 11C,D). A negative correlation was found between C_cr_ and LPO in plasma at 4 weeks after nephrectomy. The correlation between urinary albumin at 12 weeks and LPO in plasma at 4 weeks was positive.

### 2.10. Correlations between Renal Functions and Cytokines 

**Figure 12:**
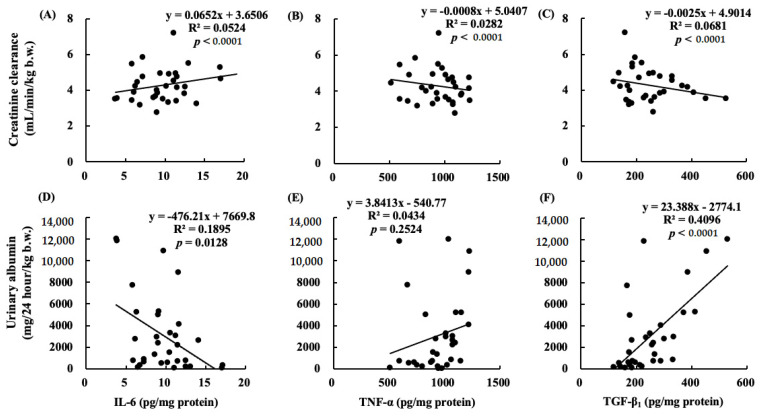
Correlations between C_cr_ and (**A**) IL-6; (**B**) TNF-α, or (**C**) TGF-β_1_. Correlations between urinary albumin at 12 weeks and (**D**) IL-6, (**E**) TNF-α, or (**F**) TGF-β_1_. The positive correlation is between creatinine clearance and (**A**) IL-6 (*p* < 0.0001). The negative correlations are between creatinine clearance and (**B**) TNF-α or (**C**) TGF-β_1_ (*p* < 0.0001). The positive correlation is between urinary albumin at 12 weeks and (**F**) TGF-β_1_ (*p* < 0.0001).

Figure 12 shows correlations between C_cr_ and cytokines in the kidney (Figure 12A–C), and urinary albumin and cytokines in the kidney (Figure 12D–F). A negative correlation was found between C_cr_ and TNF-α or TGF-β_1_. A positive correlation was found between urinary albumin at 12 weeks and TGF-β_1_.

## 3. Discussion

The purpose of this study was to assess the effects of the dietary intake of different kinds of PUFA on the renal function in chronic renal failure.

The results of the renal function analysis showed that C_cr_ decreased due to chronic renal failure. However, there were no significant differences among the different PUFAs. Therefore, we suggest that the dietary intake of PUFAs could not improve the filtration process at the glomeruli. Renal disorders increase the urinary protein excretion [17]. Our results revealed that the urinary albumin levels were increased following nephrectomy. Moreover, we found that by different kinds of dietary PUFAs attenuated the increase in the urinary albumin excretion. While the ARA and DHA groups both decreased the urinary albumin, the greatest attenuation of urinary albumin was observed in the ARA + DHA group. Urinary albumin excretion causes glomerular diseases such as podocyte injury, glomerulosclerosis by mesangial hypertrophy, and dysfunction of vascular endothelial cells [18]. The glomerular filtration barrier consists of three layers: the glomerular epithelium, the basement membrane, and slit diaphragms. The slit diaphragms are formed by the foot processes of podocytes, and they prevent the passage of proteins into the urinary filtrate [19]. Therefore, as a result of podocyte injury the slit diaphragms break and urinary albumin excretion occurs. Podocytes can be observed an electron microscope; however, we did not observe the podocytes. In the future, we will need to observe the podocytes using electron microscopy and assess the effects of ARA and DHA on podocytes. Glomerular hypertrophy is known to occur following glomerular injury. We used H&E staining to assess the overall coronal section, calculated the area of the inner and area of outer levels of glomeruli, and assessed the glomerular hypertrophy. PAS staining was used to evaluate glomerulosclerosis, and PAM staining was used to evaluate the injury to the glomerular basement membrane and mesangial cells. Glomerular hypertrophy, glomerulosclerosis, glomerular basement membrane, and mesangial cells did not significantly different among the five groups. Therefore, we suggest that the overall coronal section, glomerulosclerosis, and injury of the mesangial cells were not affected by the consumption of different kinds of PUFAs, and kidney tissues except mesangial cells were affected.

It has also been reported that an increase in oxidative stress reduces renal functions [20]. The results of our study showed that the levels of ROS and ONOO^−^ in the kidney decreased with the intake of ARA and DHA at 16 weeks after nephrectomy (Figure 7 and Figure 8). However, ROS, ONOO^−^ and LPO at 16 weeks after nephrectomy were not correlated with renal functions. We found that the LPO levels decreased with the intake of ARA and DHA in the plasma at 4 weeks after nephrectomy (Figure 9B). Moreover, there was a negative correlation between LPO levels and creatinine clearance, and a positive correlation between the LPO levels, but there was positive correlation between the LPO levels and the urinary albumin level at 4 weeks after nephrectomy was observed (Figure 11). Based on these results, we suggest that ARA and DHA could suppress oxidative stress in the early stage of renal failure and inhibit the progression of renal failure. However, the ROS, ONOO^−^, and LPO levels in the kidney were not measured at the early stage of renal failure because the kidneys did not collect at that time. In the future, we need to confirm that oxidative stress indeed increases during the early stage of renal failure.

Moreover, ONOO^−^ is involved in vascular endothelial dysfunction [21]. ONOO^−^ is produced by the reaction of nitric oxide (NO) and oxidative stress. It is known to cause vascular endothelial dysfunction due to NO deficiency; the increase in ONOO^−^ levels decreased the bioavailability of NO in CKD. The decrease in ONOO^−^ by ARA + DHA may be related to attenuated vascular endothelial dysfunction in the kidney and attenuated urinary albumin excretion. However, we did not assess the vascular endothelial dysfunction in the kidneys. Additional research is needed to determine whether urinary albumin excretion is increased by vascular endothelial dysfunction due to oxidative stress induced by the renal failure.

Body weight changes at 16 weeks after nephrectomy were not statistically different among the five groups. In contrast, the kidney weight decreased in the nephrectomy group. In the present study, although five-sixths of the kidneys were removed, the weight of the remaining kidney was 60% compared to that of intact kidneys, suggesting that the remaining kidneys were regenerated, which would compensate for kidney function. Additional research is required to confirm whether some growth factors that regenerate the kidney are affected by ARA or DHA.

Our results revealed that the protein levels decreased following nephrectomy and were recovered by the DHA group, suggesting that the levels of the biological components other than protein had increased.

An increase in the oxidative stress and inflammation by triglyceride levels in the kidneys has been previously reported [22,23]. Our results showed that triglycerides levels decreased after nephrectomy. Not only did the triglyceride levels deceased in the ARA group, but it was also recovered by feeding the rats different kinds of PUFAs.

Long-term administration of ARA to healthy older rats did not increase the production of oxidative stress and inflammatory cytokines in the kidneys, in contrast to the increase in the ARA-derived eicosanoids [3]. ω-3 PUFA-derived resolvins (Rvs) and protectins (PDs) inhibit neutrophil infiltration into the injured kidneys, block toll-like receptor-mediated inflammatory activation of macrophages, and mitigate renal function. The inhibitory effects of ω-3 PUFAs on renal injury associated with the metabolic syndrome have been reported and increased inflammation has been shown to reduce renal function [24]. Our results showed that the TNF-α and TGF-β_1_ levels in the kidneys increased following nephrectomy and this increase was negatively correlated with creatinine clearance. In contrast, the increase in TGF-β_1_ levels due to chronic renal failure was positively correlated with the urinary albumin level (Figure 12). TGF-β_1_ not only induces anti-inflammatory cytokines but also induces fibrosis [25]. We suggested that ARA + DHA decreased TGF-β_1_ levels in kidney and attenuated kidney fibrosis. However, mesangial cells and glomerular sclerosis were not assessed by PAS staining. In the future, we will necessary to assess mesangial cells and glomerular sclerosis by Masson’s trichrome staining.

In conclusion, the results of the present study indicate that ARA and DHA may have suppressive effects on the progression of renal failure. One possible mechanism is the suppression of oxidative stress in the early stages of renal failure. Diets rich in ARA and DHA were found to suppress the oxidative stress in early renal failure and the inflammation at 16 weeks after renal failure. Therefore, we suggest that different suppression mechanisms by ARA + DHA are involved in the relationship between oxidative stress and inflammation. Future studies are required to clarify these mechanisms.

## 4. Materials and Methods

### 4.1. Animals

All experiments were carried out in accordance with the Guidelines for Animal Experimentation of Josai University and were approved by the Animal Care and Use Committee of the same institution (H28006, approval on 1 April 2017). The study was performed in compliance with the Guiding Principles for the Care and Use of Animals in the Field of Physiological Science of the Physiological Society of Japan. Male Sprague DAWLEY (SD, 6 weeks old) rats were used in this study. The rats were purchased from Sankyo Labo Service Corporation (Tokyo, Japan), and housed in a room under controlled temperature (25 ± 2 °C), humidity (60 ± 5%), and light–dark cycle (7:00–19:00).

### 4.2. Diets

Diets were supplied by Suntory Wellness Ltd (Kyoto, Japan). They were modified fatty acid compositions of diets based on AIN-76A, in which the adjusted ratio of ω-6 PUFA to ω-3 PUFA is 2 to 1 and the PUFA to SFA to MUFA ratio is 1 to 1 to 1. The fatty acid composition of the diets is shown in Table 4.

### 4.3. Nephrectomy

Rats were randomly assigned into 4 groups; all groups were fed ad libitum with water and one of the four: control, ARA, DHA, and ARA + DHA-containing diets for 4 weeks. Then five-sixths of the kidneys were removed from each rat. The rats were anesthetized using a mix of three anesthetic types: medetomidine/midazolam/butorphanol (0.5/5.0/2.5 mg/mL). First, two-thirds of the left-side kidney were removed and then, after 2 weeks, the whole right-side kidney was removed.

### 4.4. Calculation of Creatinine Clearance

Creatinine clearance was calculated using the following equation:

Creatinine clearance = Ucr × V/Pcr × b.w.

where Pcr is the creatinine level in plasma (mg/dL), Ucr is the creatinine level in urine (mg/dL), V is the urine volume (mL/min), and b.w. is the body weight (kg).

### 4.5. Sample Collection

Every 4 weeks, rats were housed in individual metabolic cages (SN-781, Shinano, Saitama, Japan) and urine and feces were separately collected for 24 h. Urine samples were cleared of debris by centrifugation. Part of each urine sample was used to calculate urinary albumin, urinary glucose, and urinary creatinine. Blood samples were collected from the tail vein after maintaining anesthesia using an anesthetic apparatus for small animals (SN-487-IT, Shinano, Tokyo, Japan). Part of each blood sample was requested for inspection of creatinine after collecting the plasma by blood centrifugation. After 16 weeks, rats were anesthetized by isoflurane after a 12 h fasting period, blood was collected from abdominal inferior vena cava and then the kidney was removed. Collected kidneys were divided into three parts: 1/3 was used to measure the kidney weight and the remaining 2/3 of the kidney were kept at −80 °C until use.

### 4.6. Histological Evaluation 

Coronal sections of kidney tissue (3 μm thick) were stained with Hematoxylin and Eosin (H&E), Periodic Acid Schiff (PAS) or Periodic Acid Methenamine-silver (PAM) and were examined using a fluorescence microscope (BZ-X700, Keyence, Osaka, Japan). Fifty glomeruli were randomly selected from each rat for histological evaluation. In HE, the overall of coronal section was evaluated after calculating area of inner/area of outer. PAS staining was used to evaluate glomerulosclerosis. PAM staining was used to evaluate glomerular hypertrophy. Grading was as follows: 1+, <30% of glomerular area was affected; 2+, 30 to 70% of glomerular area was affected, and 3+, >70% of glomerular area was affected.

### 4.7. Sample Preparation 

Kidneys were homogenized in 4 bed volumes of PBS. The homogenate was aliquoted and kept at −30 °C for further analysis.

### 4.8. Analysis of Fatty Acids Composition

#### 4.8.1. Sample Preparation for Gas Chromatography

For analysis of the fatty acid composition in total kidney, 0.005% BHT/Methanol and tricosanoic acid (TCA) as internal standard, were added to each kidney homogenate and then kept at −30 °C. Next, the samples were heated at 98 °C for 1 h after addition of acetyl chloride. Samples were shaken for 3 min after the sequential addition of 0.5 M sodium hydroxide/10% sodium chloride and octane. Then, samples were centrifuged at 950× *g* for 10 min at 20 °C and the top layer was collected. The fatty acid composition of kidneys was measured by gas chromatography (GC).

#### 4.8.2. GC Analysis

Fatty acid composition of kidneys was measured using the GC-2014 (Shimadzu, Kyoto, Japan) equipped with a flame ionization detector and an automatic sampler (AOC-20i, Shimadzu). GC was performed using a capillary column (DB-WAX 30 m × 0.53 mm × I.D 3 μm); for sample injection the split method was used with a split ratio of 10.0; for the carrier gas, nitrogen gas was used. GC was set up at 250 °C, initially maintained at 55 °C for 5 min. The temperature rose to 230 °C within 17 min and it was maintained for 17 min. The run time per sample was set to 39 min.

### 4.9. Quantification of Protein Level in the Kidneys

To quantify the protein amount in the kidneys, equal volume of 0.1 M sodium hydroxide was added to the kidney homogenate. The protein concentration was determined with the BCA Protein Assay Kit (Takara, Shiga, Japan). After 1 hour of incubation at 95 °C using Applied Biosystems (Thermo Scientific, MA, USA), mixed each sample with working solution of kit in each well of 96-well clear plate. The micro plate was incubated for 30 min at 37 °C and the absorbance (562 nm) was measured using SpectraMax M2e (Molecular Devices, San Jose, CA, USA) after. Protein concentration was calculated from the absorbance reading using a linear calibration curve of bovine serum albumin (BSA) as an internal standard.

### 4.10. Quantification TG in Plasma and Kidneys 

The amount of triglycerides was quantified on the total lipids extracted from the kidneys using the Bligh–Dyer extraction method [26]. After drying them down by N2 gas, total lipids were dissolved in at a ratio of total lipids to isopropyl alcohol and triton-100, 9 to 1. TG in plasma were determined using the TG assay kit (Wako Diagnostics, Osaka, Japan) according to manufacturer’s instructions and measured using a spectrophotometer (UV mini-1240, Shimadzu).

### 4.11. Analysis of Oxidative Stress Status

#### 4.11.1. ROS Levels in the Kidney

To measure the reactive oxidation status (ROS) as an index of the oxidative stress in the kidneys, 0.005% BHT/PBS and 1 mM 2′,7′- dichlorofluorescein diacetate (DCF-DA)/0.005% BHT/PBS were added to kidney homogenate, and the reaction was promoted by 15 min incubation at 37 °C. Next, the homogenates were centrifuged for 10 min (10,000× *g* at 4 °C) and then the supernatant was removed. The pellets were dissolved in 0.005% BHT/PBS and processed using ultrasonication (US CREANER USK-4K, As one, Osaka, Japan) on ice for 5 min. The samples were then loaded on a 96-well microplate (Micro plate 96 well black, Greiner, Germany) for fluorescence measurement (excitation; 494 nm, mission; 520 nm) using SpectraMax M2^e^ at 0, 10, 30, and 60 min. The amount of DCF produced in the samples was calculated from the fluorescence reading using a linear calibration curve of DCF as internal standard substance. 

#### 4.11.2. ONOO^−^ levels in the Kidney

To measure ONOO^−^ as an index of the oxidative stress in the kidneys, 0.005% BHT/PBS and 1 mM 2′,7′- dichlorodihydrofluorescein diacetate (DCFH-DA)/0.005% BHT/PBS were added to the kidney homogenate, and the reaction was promoted by incubation at 37 °C for 15 min. Next, the homogenates were centrifuged for 10 min (10,000× *g* at 4 °C) and then the supernatant was removed. The pellets were dissolved in 0.005% BHT/PBS and were further proceeded using ultrasonication on ice for 5 min. The samples were then loaded on a 96-well microplate (Micro plate 96 well black, Greiner, Germany) for fluorescence measurement (excitation, 494 nm; emission, 520 nm) using SpectraMax M2^e^ every 0, 10, 30, and 60 min. The amount of DCF produced in the samples was calculated from the fluorescence reading using a linear calibration curve of DCF as internal standard substance.

#### 4.11.3. LPO Levels in Plasma and Kidney

For measuring the amount of LPO in blood at 4 and 16 weeks after nephrectomy, collected blood samples were centrifuged for 10 min (1000× *g* at 4 °C) and the supernatant was stored at −80 °C. After the samples were stabled for one month, the TBARS assay kit was used according to manufacturer’s instruction (Cayman Chemical Company, MI, USA).

For measured the amount of LPO in the kidneys, RIPA buffer was added in the kidney homogenates and they were sonicated for 15 s at 40 V on ice. Then they were centrifuged for 10 min (1600× *g* at 4 °C) and the supernatant was stored at −80 °C. TBARS assay kit was used according to manufacturer’s instruction. The sample fluorescence was measured using SpectraMax M2^e^ at excitation, 530 nm; emission, 570 nm; cut off, 550 nm.

### 4.12. Statistical Analysis

All data are expressed as the mean ± standard errors. Data were analyzed with a one-way ANOVA with Tukey’s Honest Significant Difference test. Differences between the groups were considered significant at *p* < 0.05. All statistical analyses were performed using JMP (JMP for MAC 13.0.0, SAS institute Japan, Tokyo, Japan).

## 5. Conclusions

Consuming ARA and DHA could potentially suppress the oxidative stress in the early stage of renal failure and could in turn suppress the progression of renal failure.

## Figures and Tables

**Table 1:** Changes in renal parameters after nephrectomy.

	Weeks	CS	Control	ARA	DHA	ARA + DHA
	0	175.9 ± 11.7	151.2 ± 4.4	149.3 ± 5.8	138.3 ± 7.6	155.7 ± 2.8
Blood glucose	4	170.1 ± 13.9	181.3 ± 6.0	180.8 ± 7.4	168.0 ± 9.1	176.8 ± 13.6
(mg/dL)	8	203.0 ± 12.0	187.0 ± 6.4	164.3 ± 8.1	177.7 ± 6.9	174.0 ± 5.8
	12	186.9 ± 5.7	174.2 ± 6.5	177.5 ± 7.0	183.5 ± 4.7	189.5 ± 7.5
	0	31.2 ± 5.0	27.2 ± 4.1	24.3 ± 4.4	20.0 ± 2.1	26.5 ± 8.1
Urinary glucose	4	37.7 ± 8.3	34.5 ± 5.3	34.3 ± 4.4	31.8 ± 4.1	33.8 ± 2.2
(mg/dL)	8	43.7 ± 5.3	41.7 ± 3.5	28.5 ± 2.8	35.0 ± 2.1	34.8 ± 1.2
	12	37.7 ± 8.6	29.5 ± 3.6	40.7 ± 6.8	37.8 ± 5.2	38.3 ± 4.3
	0	7.7 ± 0.2 ^a^	4.9 ± 0.2 ^b^	4.7 ± 0.3 ^b^	4.3 ± 0.2 ^b^	4.6 ± 0.3 ^b^
Creatinine clearance	4	5.9 ± 0.2	5.2 ± 0.2	5.1 ± 0.2	4.5 ± 0.2	4.3 ± 0.2
(mL/min/kg b.w.)	8	5.9 ± 0.3 ^a^	4.3 ± 0.3 ^b^	4.5 ± 0.2 ^b^	3.9 ± 0.2 ^b^	4.6 ± 0.2 ^b^
	12	5.1 ± 0.2 ^a^	3.9 ± 0.2 ^b^	4.1 ± 0.3 ^b^	3.8 ± 0.3 ^b^	3.9 ± 0.3 ^b^
	0	0.1 ± 0.0	0.2 ± 0.1	0.1 ± 0.0	0.1 ± 0.0	0.1 ± 0.0
Urinary albumin	4	0.1 ± 0.9	2.1 ± 0.9	1.4 ± 1.0	0.6 ± 0.0	0.6 ± 0.3
(g/24 hour/kg)	8	0.2 ± 0.1 ^a^	5.0 ± 1.6 ^b^	1.9 ± 0.8 ^a^	1.3 ± 0.1 ^a^	1.2 ± 0.5 ^a^
	12	0.6 ± 0.2 ^a^	11.4 ± 3.0 ^b^	5.9 ± 2.6 ^a,b,c^	5.6 ± 0.3 ^a,b,c^	3.0 ± 1.2 ^a,c^

Values are presented as the mean ± SEM (*n* = 6–9). ^a–c^, Differences among four different time points by ANOVA and Tukey HSD test (*p* < 0.01).

**Table 2:** Fatty acid composition of total lipid in plasma.

(%)	CS	Control	ARA	DHA	ARA + DHA
C16:0	26.3 ± 1.9	27.8 ± 0.9	23.7 ± 4.1	27.9 ± 0.7	27.7 ± 0.3
C16:1	0.7 ± 0.4	0.4 ± 0.1	0.6 ± 0.4	0.4 ± 0.0	0.3 ± 0.1
C18:0	10.4 ± 1.0	11.5 ± 0.5	11.2 ± 0.8	12.0 ± 0.8	9.7 ± 0.3
C18:1	21.0 ± 1.8 ^a^	18.2 ± 1.4 ^a,b^	16.0 ± 1.1 ^a,b^	15.3 ± 0.6 ^b^	13.1 ± 0.4 ^b^
C18:2ω-6	10.7 ± 0.9 ^b,c^	12.5 ± 0.3 ^a,b^	8.4 ± 0.6 ^c^	13.7 ± 0.3 ^a^	8.6 ± 0.3 ^c^
C20:0	1.1 ± 0.2	0.9 ± 0.3	1.0 ± 0.4	0.4 ± 0.2	0.3 ± 0.1
C20:4ω-6	26.5 ± 5.8	25.3 ± 1.9	36.3 ± 3.0	22.7 ± 1.4	34.2 ± 0.7
C20:5ω-3	0.0 ± 0.0	0.0 ± 0.0	0.3 ± 0.2	0.0 ± 0.0	0.4 ± 0.2
C22:0	0.2 ± 0.2	0.0 ± 0.0	0.0 ± 0.0	0.0 ± 0.0	0.0 ± 0.0
C22:6ω-3	3.2 ± 0.3 ^b^	3.4 ± 0.7 ^b^	2.4 ± 0.5 ^b^	7.6 ± 0.4 ^a^	5.7 ± 0.4 ^a^
ω-6/ω-3	12.2 ± 6.2	5.0 ± 1.0	10.1 ± 2.2	3.1 ± 0.3	5.7 ± 0.4

Palmitic acid; C16:0, palmitoleic acid; C16:1, stearic acid; C18:0, oleic acid; C18:1, linoleic acid; C18:2ω-6, arachidic acid; C20:0, arachidonic acid; C20:4ω-6, docosanoic acid; C20:5ω-3, docosanoic acid; C22:0, eicosapentaenoic acid; C22:6ω-3, ratio of ω-6 and ω-3; ω-6/ω-3.Values are presented as the mean ± SEM (*n* = 6–9). ^a–c^, by ANOVA and Tukey HSD test (*p* < 0.01).

**Table 3:** Fatty acid composition of total lipid in kidney.

(%)	CS	Control	ARA	DHA	ARA + DHA
C8:0	0.3 ± 0.3	0.0 ± 0.0	0.0 ± 0.0	0.0 ± 0.0	0.1 ± 0.1
C10:0	0.8 ± 0.3	0.7 ± 0.0	0.4 ± 0.0	0.7 ± 0.1	0.6 ± 0.1
C12:0	0.5 ± 0.4	0.1 ± 0.0	0.1 ± 0.0	0.1 ± 0.1	0.3 ± 0.1
C14:0	1.0 ± 0.4	0.7 ± 0.1	0.7 ± 0.1	0.6 ± 0.1	1.0 ± 0.2
C16:0	26.0 ± 0.8	27.7 ± 0.3	27.6 ± 0.4	27.3 ± 0.4	28.2 ± 0.9
C16:1	1.7 ± 0.2	1.5 ± 0.2	2.0 ± 0.3	1.4 ± 0.3	2.4 ± 0.6
C18:0	16.9 ± 0.7	16.5 ± 0.7	16.9 ± 0.7	17.0 ± 0.6	15.6 ± 1.3
C18:1	15.6 ± 1.2	16.3 ± 1.3	16.5 ± 1.4	14.4 ± 1.4	16.1 ± 3.2
C18:2ω-6	8.0 ± 0.3 ^b^	8.3 ± 0.2 ^b^	5.2 ± 0.3 ^c^	9.7 ± 0.2 ^a^	6.1 ± 0.2 ^c^
C18:3ω-3	0.8 ± 0.2	0.7 ± 0.1	0.8 ± 0.1	0.3 ± 0.1	0.6 ± 0.1
C20:0	1.0 ± 0.4	0.7 ± 0.1	0.7 ± 0.1	0.7 ± 0.1	0.8 ± 0.2
C20:4ω-6	18.9 ± 1.3	17.9 ± 1.1	22.3 ± 1.3	17.0 ± 1.0	19.6 ± 2.3
C22:0	2.5 ± 0.3 ^a,b^	2.7 ± 0.2 ^a,b^	1.8 ± 0.1 ^b^	3.5 ± 0.2 ^a^	2.3 ± 0.3 ^b^
C24:0	3.1 ± 0.3	2.8 ± 0.2	2.7 ± 0.2	3.1 ± 0.2	2.7 ± 0.3
C22:6ω-3	2.7 ± 0.3 ^b,c^	3.2 ± 0.2 ^a,b,c^	2.2 ± 0.2 ^c^	4.2 ± 0.2 ^a^	3.7 ± 0.5 ^a,b^
ω-6/ω-3	8.0 ± 0.8 ^a,b^	6.7 ± 0.3 ^b,c^	8.9 ± 0.2 ^a^	6.1 ± 0.2 ^c^	6.0 ± 0.3 ^c^

Caprylic acid; C8:0, capric acid; C10:0, lauric acid; C12:0, myristic acid; C14:0, palmitic acid; C16:0, palmitoleic acid; C16:1, stearic acid; C18:0, oleic acid; C18:1, linoleic acid; C18:2ω-6, α-linolenic acid; C18:3ω-3, arachidic acid; C20:0, arachidonic acid; C20:4ω-6, docosanoic acid; C22:0, lignoceric acid; C24:0, eicosapentaenoic acid; C22:6ω-3, ratio of ω-6 and ω-3; ω-6/ω-3.Values are presented as the mean ± SEM (*n* = 6–9). ^a–c^, by ANOVA and Tukey HSD test (*p* < 0.01).

**Table 4:** Fatty acid composition of diets (%) based on AIN-76A.

(%)	Control	ARA	DHA	ARA + DHA
PLA (16:0)	27.6	27.2	27.3	28.1
STA (18:0)	4.2	4.6	4.4	4.8
OLA (18:1)	31.5	29.8	29.9	28.8
LA (18:2ω-6)	22.3	17.7	21.7	16.4
ALA (18:3ω-3)	11.3	11.6	6.2	5.8
ARA (20:4ω-6)	0.0	4.1	0.2	0.8
EPA (20:5ω-3)	0.0	0.0	0.8	0.8
DHA (22:6ω-3)	0.0	0.0	4.0	4.0
SFA	33.1	34.2	33.4	35.6
MUFA	31.9	30.3	31.3	30.2
PUFA	34	34.6	33.8	32.7
ω-6/ω-3	1.98	1.91	2.01	2.01

ALA, α-linoleic acid; ARA, arachidonic acid; DHA, docosahexaenoic acid; EPA, eicosapentaenoic acid; LA, linoleic acid; MUFA, monounsaturated fatty acid; OLA, oleic acid; PLA, palmitic acid; PUFA, polyunsaturated fatty acid; SFA, saturated fatty acid; STA, stearic acid.

## Data Availability

The data presented in this study are available on request from the corresponding author.
